# Supplementary material for: Comparative multiomics analysis of cell physiological state after culture in a basket bioreactor
Source: Sci Rep. 2022 Nov 23;12:20161. doi: 10.1038/s41598-022-24687-4 (PMC9686226; doi:10.1038/s41598-022-24687-4)
Supplement: Supplementary file 1 — Supplementary Information 1. [file 41598_2022_24687_MOESM1_ESM.zip › raw data/Transcriptomic raw data/6.Enrichment/2.KEGG/TE1vsTC1/DOWN/src/csab00600.html]

csab00600


- csab:103233234

- Down regulated genes

103233234(-2.15784910790094)

- csab:103224742

- Down regulated genes

103224742(-1.30659810321941)

- csab:103224742

- Down regulated genes

103224742(-1.30659810321941)

- csab:103224742

- Down regulated genes

103224742(-1.30659810321941)

Close
